# Synchrony in the periphery: inter-subject correlation of physiological responses during live music concerts

**DOI:** 10.1038/s41598-021-00492-3

**Published:** 2021-11-17

**Authors:** Anna Czepiel, Lauren K. Fink, Lea T. Fink, Melanie Wald-Fuhrmann, Martin Tröndle, Julia Merrill

**Affiliations:** 1 Max Planck Institute for Empirical Aesthetics, Frankfurt am Main, Germany; 2 Max Planck - NYU Center for Language, Music, & Emotion (CLaME), New York, USA; 3 Zeppelin University, Friedrichshafen, Germany; 4 Institute of Music, University of Kassel, Kassel, Germany

**Keywords:** Neuroscience, Physiology, Psychology

## Abstract

While there is an increasing shift in cognitive science to study perception of naturalistic stimuli, this study extends this goal to naturalistic contexts by assessing physiological synchrony across audience members in a concert setting. Cardiorespiratory, skin conductance, and facial muscle responses were measured from participants attending live string quintet performances of full-length works from Viennese Classical, Contemporary, and Romantic styles. The concert was repeated on three consecutive days with different audiences. Using inter-subject correlation (ISC) to identify reliable responses to music, we found that highly correlated responses depicted typical signatures of physiological arousal. By relating physiological ISC to quantitative values of music features, logistic regressions revealed that high physiological synchrony was consistently predicted by faster tempi (which had higher ratings of arousing emotions and engagement), but only in Classical and Romantic styles (rated as familiar) and not the Contemporary style (rated as unfamiliar). Additionally, highly synchronised responses across all three concert audiences occurred during important structural moments in the music—identified using music theoretical analysis—namely at transitional passages, boundaries, and phrase repetitions. Overall, our results show that specific music features induce similar physiological responses across audience members in a concert context, which are linked to arousal, engagement, and familiarity.

## Introduction

While there is an increasing shift in cognitive science to study human perception of naturalistic stimuli (e.g., real-world movies or music^1^), such research is still required in more naturalistic contexts. A concert setting provides one promising context to which research focusing on perception and experience of music can be extended; not only does it afford one possible naturalistic setting for music listening, but also live performances can evoke stronger emotional responses^2–4^ and offer a more immersive experience^5,6^. Although brain imaging techniques can implicitly measure undisturbed (i.e., without behavioural ratings) naturalistic musical perception as it evolves^7–10^, these methods lack applicability in a wider range of typical listening situations; therefore, more portable methods for measuring continuous responses such as motion capture^11^ or mobile measurement of peripheral physiology^12,13^ are required. As our interest lay in the musical experience within the context of a Western art music concert—in which listeners are typically still^6,14^—we focused on physiological responses of the autonomic system (ANS).

Previous research shows that certain physiological signatures indicative of a momentary ANS activations, regarding (phasic) skin conductance response (SCR, i.e., sweat secretion), heart rate (HR), and respiration rate (RR) as well as responses of facial muscles (electromyography [EMG] measurement), are related to orientation responses^15,16^ and affective processing^17–20^. Event-related changes in SCR, HR, RR, and EMG—reflecting a startle^21–23^ or orienting response^24^—have been associated with pitch changes^25,26^ and tone loudness (the louder the sound, the greater the SCR amplitude^27,28^), as well as deviations in timbre, rhythm, and tempo^28^. In other words, such signatures may be a response to novelty in stimuli.

Additionally, physiological responses have been shown to occur in response to arousing acoustic features^17–20^. For example, faster and increasing tempi are associated with greater arousal (reflecting emotions such as happiness) in the music^29–32^, which correspond to increased HR^32–35^, RR^36^, and SCR^28,32,35,37,38^ in a listener. Slower-paced music (reflecting low arousal emotions such as sadness) reduces HR, RR, and SCR^36,39,40^. Timbral features, such as brighter tones and higher spectral centroid, are associated with higher arousal^29,41,42^, which correlates somewhat to RR and SCR^35,43,44^. Loudness is positively correlated with arousal^29,45,46^, and, correspondingly, changes in SCR^47,48^ and HR^29,49^. Ambiguous harmony—operationalised as unexpected harmonic chords (i.e., out-of-key chords in place of tonic chords) and unpredictable notes (i.e., surprising notes within a predictable melodic sequence)—as well as dissonance may also be perceived as tense/arousing^50,51^ and lead to event-related increases in SCR^50,52,53^. Similarly, new or unprepared harmony, enharmonic changes, and harmonic acceleration to cadences have been found to evoke chills^54^, which are also related to increased SCR, HR, and RR when listening to music^55–60^^.^. Overall, this shows that peripheral physiology is related to arousing acoustic features, namely faster tempos, harmonic ambiguity, loudness, and (to some extent) timbral brightness. Importantly, it seems that increases in self-reported arousal and physiological measures are time-locked together in an event-related fashion^13,61^, suggesting that an increase in reported arousal is simultaneously reflected by increased ANS responses.

It is also worth noting that some ANS responses may be modulated by musical style. Previous studies found that HR increases with faster tempo in Classical music, but decreases with faster tempo in rock music^34^. HR is overall lower in atonal, compared to tonal music, independent of the emotional characteristics of the music^62^.

Although previous research generally supports the idea that specific physiological features are associated with specific musical features and styles, some of these studies (for reasons of experimental control) have carefully chosen and cut or constructed stimuli to have little variability in acoustic features (e.g., they use a constant tempo and normalise loudness). However, more research into full-length naturalistic stimuli—which typically have a rich dynamic variation of interdependent features—is required^1^.

While previous work using naturalistic music has correlated neural and physiological responses to dynamically changing acoustic features^8,10,43,63^, or extracted epochs based on information content in the music^13^, perhaps a more robust way to identify systematic responses to naturalistic stimuli is via synchrony of responses^12,64–67^, in particular via inter-subject correlation (ISC, see review^68^). This method—in which (neural) responses are correlated across participants exposed to naturalistic stimuli^69^—is based on the assumption that signals not related to processing stimuli would not be correlated. Such synchrony research has demonstrated that highly similar responses occur across subjects when exposed to naturalistic films^69–73^, spoken dialogue^74–76^ and text^77,78^, dance^79^, and music^7–9,80^, strongly suggesting that highly reliable and time-locked responses can be evoked by (seemingly uncontrolled) complex stimuli (for a review see^81^). Although ISC in functional magnetic resonance imaging (fMRI) studies can identify regions of interest (ROIs) for further analysis^69^, ISC can also assess which kind of feature(s) within dynamically evolving stimuli evoke highly correlated responses.

In response to auditory stimuli, higher synchronisation (operationalized via ISC) of participants’ responses has been associated with emotional arousal, structural coherence, and familiarity. In terms of emotional arousal, higher correlation coefficients of electroencephalography (EEG)^71^, SCR, and respiration^82^ (as well as higher hemodynamic activity in ROIs that had highly correlated activity between participants’ fMRI^81^) coincided with moments of high arousal in films, such as a close-up of a revolver^71^, gun-shots or explosions^69^, as well as close-ups of faces and emotional shakiness in voice^82^. Additionally, physiological synchrony between co-present audience members of movie viewings and theatre performance correlate with convergence of emotional responses and evaluation^12,72^. Regarding structural coherence, ISC is higher when listening to original, compared to phase-scrambled, versions of music^7,80^ and spoken text^78^. Cardiovascular and respiratory synchrony (calculated here by Generalised Partial Directed Coherence) was lower when audiences listened to music with complex auditory profiles^64^, further suggesting physiological synchrony may be linked to structural coherence. ISC may additionally reflect familiarity and engagement: it is higher when listening to familiar musical styles, compared to unfamiliar styles; though upon repeated presentation, ISC drops with repetitions of familiar (but not unfamiliar) musical style^9^. However, many of these findings come from neural and laboratory contexts with mostly general descriptions of the stimuli. Thus, further research is required to deepen our understanding of music and ISC by investigating ISC with more portable methods such as physiology and exploring which musical features—characterised by a more comprehensive analysis—can evoke synchronised physiological responses in more naturalistic listening situations.

**Figure 1:**
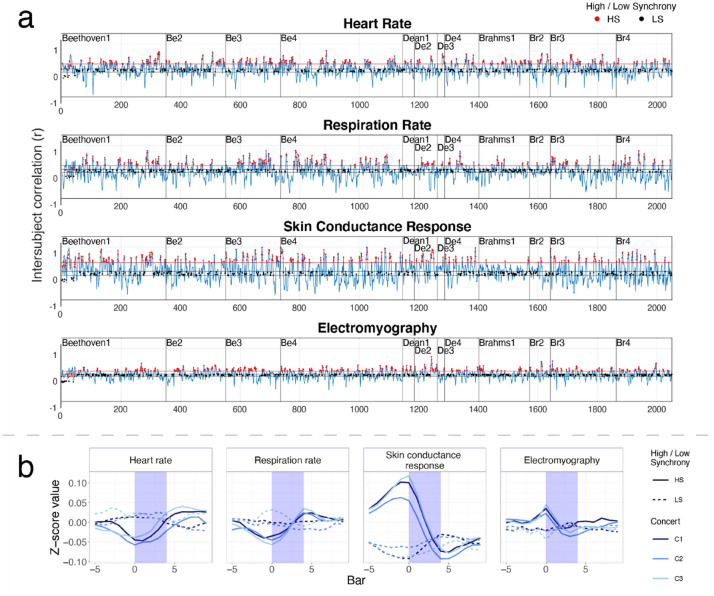
Inter-subject correlation (ISC) across concerts and bars of high- and low-synchrony. (**a**) ISC time courses for heart rate (HR, row 1), respiration rate (RR, row 2), skin conductance response (SCR, row 3), and electromyography activity of zygomaticus major (‘smiling’) muscle (EMG, row 4) for concert 1. Moments of high and low synchrony are marked with red and black dots, respectively. Red lines signify the 20th percentile threshold, while black lines signify the 20th percentile centred around *r* = 0. (**b**) Average high synchrony (HS) versus low synchrony (LS) of each physiological measure and concert. Moments of high and low synchrony are marked with solid and dotted lines, respectively. Four musical bars precede (− 4 to 0) and follow (4–8) correlation windows (highlighted in blue box) with high ISC value starting from the first bar of correlation (bar0) to last bar of correlation window (bar4).

The overarching aim of the current study was to explore which musical feature(s) evoke systematic physiological responses during undisturbed, naturalistic music listening in a concert context. Participants attended one of three chamber music concerts, with live performances of string quintets by Beethoven (1770–1827), Dean (1961–), and Brahms (1833–1897) (four movements each), showcasing different musical styles (Viennese Classical, Contemporary, and Romantic, respectively) with varying tempo, tonality, compositional structure, character, and timbre. Psychological (emotion and absorption) ratings were collected from a short (2-minute) questionnaire immediately after each movement, and familiarity ratings were collected after each piece. Continuous physiological responses were measured throughout the concert, from which SCR, HR, RR, and EMG activity of 98 participants (Concert 1 [C1]: 36, C2: 41, C3: 21) was extracted. For each audience, ISC was calculated over a sliding window (5 musical bars long) for each physiological measure, representing the degree of collective synchrony of physiological responses over the time-course of each musical stimulus (see Fig. 1a). High and low physiological synchrony were operationalised based on criteria from Dmochowksi et al.^71^. Windows containing ISC values in the highest 20th percentile represented high synchrony (HS) windows, while windows with correlation ISC values in the 20th percentile centred around *r* = 0 (that is, lowest correlation values) represented low synchrony (LS) windows.

To characterise the music, quantitative values of low- and medium-level features as well as detailed descriptions of high-level, structural features were obtained using inter-disciplinary approaches of acoustic signal and score-based analyses, respectively. Acoustic features most commonly associated with orientation and arousal physiological responses (as described above) were extracted. Instantaneous tempo was calculated using inter-onset intervals (IOIs) between each beat to represent the speed. Root mean square (RMS) energy was computationally extracted to represent loudness^46^ of the music. As the centroid of the spectral distribution of an acoustic signal has been shown to be a main contributor to perception of timbre^83^ and timbral brightness^84,85^, spectral centroid was computationally extracted (with higher values representing brighter timbre). Key clarity over time was calculated using an algorithm that correlates pitch class profiles (of the acoustic signal) with pre-defined key profiles (see^86–88^). The correlation coefficient associated with the best-matched key represents a quantitative measure of clarity of key, which is highly (positively) correlated with perceptual ratings of key clarity (i.e., the reverse of harmonic ambiguity)^8^. As we used naturalistic music, it was also important to consider stylistic and structural features of the music which cannot be analysed quantitatively (yet). Therefore, we prepared a detailed music theoretical analysis, indicating harmonic progressions, thematic and motivic relations, phrase rhythm, formal functions, and the overall repetition schema of the pieces^89,90^.

Based on the assumptions that ISC can identify systematic responses during naturalistic stimuli perception, and that certain physiological signatures and synchrony are related to musical features and self-reported states, the following hypotheses drove our research: (1) windows of high physiological synchrony—identified by ISC analysis—represent systematic physiological responses that are typically associated with event-related arousal responses; (2) highly correlated physiological responses during naturalistic music listening in a live concert context can be predicted by quantitative values of typically arousing acoustic features (higher RMS energy and spectral centroid, faster tempo, and lower key clarity), which may be modulated by the different styles. Additionally, we were (3) interested in the relations between ISC and higher-level musical features, such as compositional structure of the music. In light of the replicability crisis^91^, we tested whether robust physiological responses to music would be consistent across repeated concert performances. Such an approach simultaneously provides data regarding the stability of using a concert as an experimental setting.

## Results

### Acoustic comparisons: differences between performances and styles

Before testing our hypotheses, we assessed whether the extracted acoustic features were comparable across performances. As the music was performed by professional musicians who were instructed to play as similarly as possible across the concerts, statistical tests confirmed our expectation that all concert performances would be acoustically similar. No significant differences occurred between performances for loudness, tempo, timbre, and length, with Pearson correlations of instantaneous tempo, timbre, and loudness between all performances reaching *r* > 0.6, *p* < 0.001 (see Supplementary Tables S1a, S1b, and S1c). This confirms that performances were comparable enough to allow for further statistical comparisons of listeners’ physiology between audiences (i.e., that observed physiological responses are not attributable to unintended differences in the performances between concerts).

Because we hypothesized that style may play a role in driving physiological differences—and that certain styles are defined by differences in acoustic features—loudness, timbre, tempo, and key clarity were compared between styles. Contrasts revealed that Dean (Contemporary) had significantly lower RMS energy compared to Brahms (Romantic, *p* < 0.05). Dean also had significantly lower key clarity compared to both Beethoven (Classical, *p* < 0.012) and Brahms (*p* < 0.001), and significantly higher spectral centroid compared to Beethoven (*p* < 0.037) and Brahms (*p* < 0.046) (see Supplementary Figure S1 and Table S2a and S2b). Although tempo did not significantly differ between the styles (all *p* > 0.327), a division of tempi distribution in Beethoven and Brahms (Supplementary Figure S1) shows a typical composition practice of contrasting faster and slower movements in Classical and Romantic styles.

#### Summary

These acoustic checks confirm that our stimuli were comparable across concerts and that they offered a rich variation of acoustic features within and between pieces.

### Physiological responses during moments of high versus low synchrony

Hypothesis 1 was assessed by comparing physiology in HS and LS windows. As shown in Fig. 1b, HR and RR were overall lower in HS compared to LS windows, confirmed by significant main effect of Synchrony for HR in all concerts and for RR in C3. Significant Synchrony by Bar interactions and contrasts for RR in C2 and C3 suggested that breathing accelerates from the onset bar (bar0) to the last bar (bar4) of the HS window (see Tables 1, 2). An HR increase also seemed to occur (see Fig. 1b), but did not withstand Bonferroni correction.

**Table 1:** ANOVA tests for linear models comparing physiology in 5 bar windows for Synchrony (HS/LS) across correlation windows in terms of Bar (0–4), calculated with the *Anova* function from the *car* package in R.

Concert	df	HR		RR		SCR		EMG	
		F	*p*	F	*p*	F	*p*	F	*p*
**C1**									
Synchrony	1	**13.316**	**< 0.001**	5.146	0.023	**103.224**	**< 0.001**	**20.044**	**< 0.001**
Bar	4	**4.138**	**< 0.001**	**5.485**	**< 0.001**	**28.969**	**< 0.001**	**5.335**	**< 0.001**
Synch. × Bar	4	3.336	0.010	2.190	0.068	**26.392**	**< 0.001**	**4.760**	**< 0.001**
**C2**									
Synchrony	1	**24.426**	**< 0.001**	8.045	0.005	**52.673**	**< 0.001**	**12.815**	**< 0.001**
Bar	4	1.743	0.137	**10.263**	**< 0.001**	**26.981**	**< 0.001**	**8.042**	**< 0.001**
Synch. × Bar	4	0.846	0.495	**6.300**	**< 0.001**	**19.372**	**< 0.001**	**5.974**	**< 0.001**
**C3**									
Synchrony	1	**10.065 **	**0.002**	**25.527**	**> 0.001**	**90.166**	**< 0.001**	**29.240**	**< 0.001**
Bar	4	3.739	0.005	**10.696**	**> 0.001**	**24.780**	**< 0.001**	**6.405**	**< 0.001**
Synch. × Bar	4	**3.041 **	**0.016**	**10.354**	**> 0.001**	**15.977**	**< 0.001**	**7.846**	**< 0.001**

Bonferroni-corrected threshold for significant effect was 0.05/12 = 0.004. Values highlighted in bold are statistically significant after Bonferroni corrections for multiple comparisons.

**Table 2:** Pairwise comparisons of linear models comparing physiology in 5 bar windows for Synchrony (HS/LS) across correlation windows in terms of Bar (0–4).

Pairwise comparison	HR					RR				
	Estimate	*SE*	*df*	*t*	*p*	Estimate	*SE*	*df*	*t*	*p*
**C1**										
HS–LS	**− 0.036**	**0.007**	**4220**	**− 5.073**	**< 0.001**	− 0.010	0.007	4000	− 1.504	0.133
HS: bar0–bar4	− 0.052	0.016	4220	− 3.315	0.042	**− 0.057**	**0.014**	**4000**	**− 3.970**	**0.003**
LS: bar0–bar4	0.016	0.016	4220	0.997	1.00	− 0.004	0.015	4000	− 0.273	1.00
**C2**										
HS–LS	**− 0.065**	**0.007**	**4225**	**− 9.676**	**< 0.001**	0.002	0.006	3705	− 0.256	0.798
HS: bar0–bar4	− 0.040	0.015	4225	− 2.415	0.710	**− 0.069**	**0.013**	**3705**	**− 5.256**	**< 0.001**
LS: bar0–bar4	− 0.003	0.015	4225	− 0.193	1.0000	0.013	0.015	3705	0.837	0.998
**C3**										
HS–LS	**− 0.028**	**0.009**	**4195**	**− 3.197**	**0.001**	**− 0.028**	**0.008**	**3810**	**− 3.518**	**< 0.001**
HS: bar0–bar4	− 0.062	0.012	4195	− 3.217	0.060	**− 0.091**	**0.017**	**3810**	**− 5.495**	**< 0.001**
LS: bar0–bar4	0.0170	0.019	4195	0.880	1.00	0.041	0.018	3810	2.248	0.424

Pairwise comparison	SCR					EMG				
	Estimate	*SE*	*df*	*t*	*p*	Estimate	*SE*	*df*	*t*	*p*
**C1**										
HS–LS	**0.071**	**0.009**	**4220**	**8.364**	**< 0.001**	**0.014**	**0.006**	**4220**	**2.505**	**0.012**
HS: bar0–bar4	**0.174**	**0.019**	**4220**	**9.161**	**< 0.001**	0.034	0.013	4220	2.683	0.33
LS: bar0–bar4	− 0.062	0.019	4220	− 3.252	0.052	− 0.010	0.013	4220	− 0.780	1.00
**C2**										
HS–LS	**0.025**	**0.007**	**4200**	**3.428**	**< 0.001**	− 0.001	0.006	4215	− 0.084	0.933
HS: bar0–bar4	**0.148**	**0.017**	**4200**	**8.924**	**< 0.001**	**0.054**	**0.012**	**4215**	**4.391**	**< 0.001**
LS: bar0–bar4	− 0.028	0.016	4200	− 1.682	1.00	− 0.010	0.012	4215	− 0.845	1.00
**C3**										
HS–LS	**0.095**	**0.010**	**4205**	**9.796**	**< 0.001**	**0.018**	**0.007**	**4215**	**2.504**	**0.012**
HS: bar0–bar4	**0.185**	**0.022**	**4205**	**8.530**	**< 0.001**	**0.061**	**0.016**	**4215**	**3.758**	**0.008**
LS: bar0–bar4	− 0.025	0.022	4205	− 1.174	1.00	− 0.030	0.016	4215	− 1.868	1.00

HS–LS denotes the overall difference between HS and LS windows. Bar0–bar4 denotes the difference between the beginning and the end of the window separately in HS and LS windows. Contrasts (Bonferroni adjusted) were calculated with *emmeans* package in R. Values highlighted in bold are statistically significant after Bonferroni corrections for multiple comparisons.

SCR and EMG activity were overall higher in HS windows, compared to LS windows, confirmed by significant main effect of synchrony for SCR and EMG in all three concerts. Significant Synchrony by Bar interactions and contrasts suggested that SCR (all concerts) and EMG activity (C2 and C3) decreased across HS windows (bar0–bar4, see Tables 1, 2). Looking at a wider range of 4 bars before and after the correlation window, it seems that ISC identifies the second half of an EMG peak amplitude and SCR peak, i.e., a momentary increase of sweat secretion (see Fig. 1b).

#### Summary

Compared to the LS moments, HS windows contained higher SCR and EMG activity, and increasing RR. Such responses correspond to a momentary activation of the sympathetic division of the ANS, and have also been associated with self-reported arousal^17–20^. These results support our first hypothesis that ISC can identify systematic, event-related physiological responses, indicative of increased arousal.

### Acoustic properties as predictors of audience synchrony

Regarding our second hypothesis, quantitative values of tempo, key clarity, spectral centroid, and RMS during high and low synchrony windows were compared using logistic regression.

#### Single physiology measures

**Figure 2:**
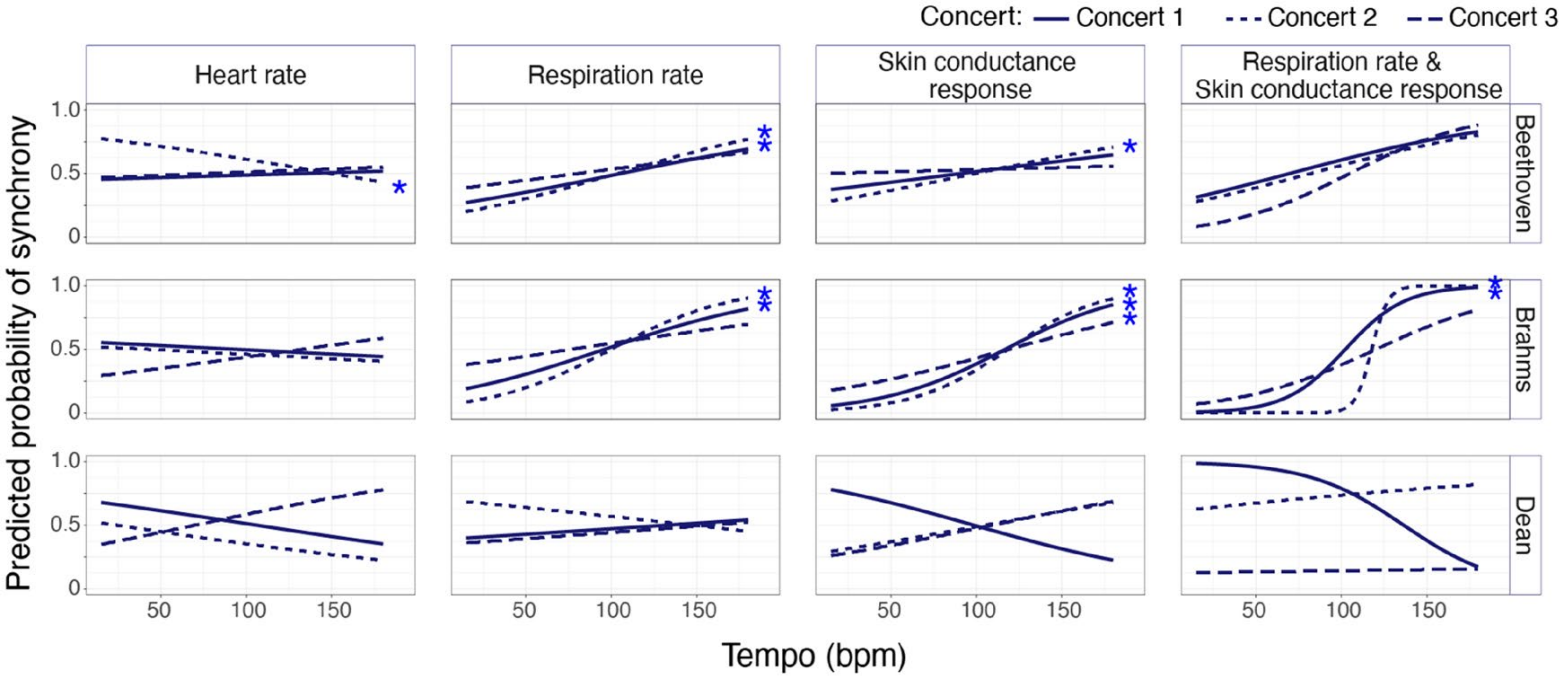
Logistic regression models with all music features (RMS, tempo, spectral centroid and key clarity) predicting high (1) versus low (0) synchrony across listeners, with probability curve of predictor tempo. Columns indicate physiological measures of interest. Rows indicate each piece performed in each of the three concerts (indicated by line style). Here, we highlight the ability of tempo to predict synchrony. For full model results for all acoustic features, see Tables 3 and 4.

Tempo significantly predicted synchronised RR arousal responses in Beethoven and Brahms C1 and C2, and significantly predicted synchronised SCR arousal responses in Beethoven C2 and Brahms all concerts, where faster tempi increased the probability of synchronised RR and SCR responses across audience members (see Table 3 and Fig. 2). Slower tempi significantly increased probability of synchronised HR arousal responses in Beethoven, but only for C2 (see Table 3). Higher RMS energy significantly increased the probability of RR synchrony in Dean C1 and SCR synchrony in Beethoven C1 (see Table 3). No significant results occurred either for EMG synchrony or for spectral centroid or key clarity.

**Table 3:** Logistic regressions for single physiological measure of respiration rate, skin conductance and heart rate synchrony per piece across all concerts (C1, C2, and C3).

	Beethoven			Dean			Brahms		
	Estimate	SE	*p* value	Estimate	SE	*p* value	Estimate	SE	*p* value
**Respiration rate**									
C1									
(Intercept)	− 1.211	0.897	0.177	1.059	2.150	0.6222	− 0.728	1.255	0.562
Tempo	**0.0110**	**0.003**	**> 0.001**	0.003	0.009	0.707	**0.018**	**0.005**	**> 0.001**
RMS	− 22.457	15.574	0.149	**212.796**	**50.117**	**> 0.001**	− 6.563	15.577	0.673
S. centroid	0.000	0.000	0.439	− 0.000	0.006	0.559	− 0.000	0.000	0.809
Key clarity	− 0.151	1.114	0.891	− 5.708	2.109	0.007	− 1.218	1.569	0.438
C2									
(Intercept)	− 0.343	1.034	0.740	2.376	1.898	0.211	− 1.451	1.297	0.263
Tempo	**0.016**	**0.004**	**> 0.001**	− 0.006	0.009	0.502	**0.028**	**0.004**	**> 0.001**
RMS	0.610	17.630	0.972	58.895	39.231	0.133	− 17.878	17.983	0.320
S. centroid	− 0.001	0.000	0.047	− 0.001	0.001	0.049	− 0.000	0.000	0.495
Key clarity	− 0.371	1.111	0.738	− 0.305	2.017	0.880	− 1.089	1.713	0.525
C3									
(Intercept)	− 1.614	0.953	0.090	− 0.320	1.521	0.833	− 0.3972	1.134	0.726
Tempo	0.007	0.003	0.016	0.004	0.007	0.537	0.008	0.003	0.0188
RMS	23.926	15.481	0.122	33.370	34.602	0.335	− 22.432	15.333	0.143
S. centroid	0.000	0.000	0.618	− 0.0002	0.001	0.628	− 0.000	0.000	0.384
Key clarity	0.947	1.187	0.425	− 0.478	1.685	0.777	0.929	1.415	0.511

	Beethoven			Dean			Brahms		
	Estimate	SE	*p* value	Estimate	SE	*p* value	Estimate	SE	*p* value
**Skin conductance**									
C1									
(Intercept)	− 1.959	0.913	0.032	3.522	1.813	0.052	− 5.518	1.334	**>** 0.001
Tempo	0.007	0.003	0.016	− 0.015	0.008	0.0551	**0.028**	**0.005**	**> 0.001**
RMS	**76.201**	**17.351**	**> 0.001**	6.880	36.553	0.851	22.717	15.291	0.137
S. centroid	0.001	0.000	0.048	0.000	0.001	0.728	0.006	0.000	0.134
Key clarity	− 0.626	1.024	0.541	− 4.674	1.795	0.009	1.6770	1.532	0.274
C2									
(Intercept)	− 1.015	0.913	0.266	− 3.901	2.091	0.061	− 5.567	1.470	**> **0.001
Tempo	**0.011**	**0.003**	**> 0.001**	0.010	0.009	0.264	**0.036**	**0.006**	**> 0.001**
RMS	17.223	15.542	0.268	29.043	43.571	0.505	44.689	16.671	0.007
S. centroid	− 0.000	0.000	0.877	0.002	0.0001	0.006	0.000	0.000	0.438
Key clarity	− 0.300	1.094	0.784	− 1.475	2.094	0.481	0.302	1.740	0.862
C3									
(Intercept)	− 0.660	0.883	0.455	0.512	1.576	0.745	− 4.412	1.257	**> 0.001**
Tempo	0.001	0.003	0.606	0.011	0.007	0.095	**0.015**	**0.004**	**> 0.001**
RMS	21.770	15.067	0.148	− 73.883	40.619	0.069	4.975	16.431	0.762
S. centroid	0.000	0.000	0.152	− 0.001	0.001	0.174	0.001	0.000	0.209
Key clarity	− 0.338	1.016	0.740	0.253	1.714	0.882	2.825	1.485	0.057

	Beethoven			Dean			Brahms		
	Estimate	SE	*p* value	Estimate	SE	*p* value	Estimate	SE	*p* value
**Heart rate**									
C1									
(Intercept)	− 0.7443	0.916	0.416	5.459	2.028	0.007	0.996	1.180	0.399
Tempo	0.002	0.003	0.566	− 0.008	0.007	0.261	− 0.003	0.003	0.343
RMS	− 25.496	16.908	0.132	− 86.004	40.332	0.033	1.825	13.783	0.895
S. centroid	0.000	0.0003	0.103	− 0.001	0.001	0.048	− 0.001	0.000	0.161
Key clarity	0.008	0.979	0.993	− 3.620	2.063	0.079	− 0.036	1.532	0.981
C2									
(Intercept)	− 0.435	0.899	0.629	0.480	1.490	0.747	0.367	1.213	0.762
Tempo	**− 0.009**	**0.003**	**0.001**	− 0.008	0.006	0.214	− 0.003	0.003	0.406
RMS	20.089	14.853	0.176	25.795	32.573	0.428	− 18.912	16.274	0.245
S. centroid	0.001	0.0003	0.009	0.0002	0.001	0.626	0.001	0.000	0.052
Key clarity	0.520	1.011	0.607	− 1.765	1.376	0.200	− 1.901	1.499	0.205
C3									
(Intercept)	2.544	0.907	0.005	1.229	1.726	0.477	− 2.139	1.1802	0.070
Tempo	0.002	0.003	0.434	0.011	0.007	0.094	0.008	0.003	0.01
RMS	− 47.392	15.412	0.002	− 68.302	39.045	0.080	4.159	14.888	0.7800
S. centroid	− 0.001	0.0002	0.029	0.001	0.001	0.307	0.000	0.000	0.761
Key clarity	− 2.087	1.065	0.050	− 0.798	1.947	0.682	1.457	1.433	0.309

Synchrony (HS = 1, LS = 0) was the dependent variable, and tempo, key clarity, loudness, and spectral centroid (s. centroid) from the HS and LS bars as continuous predictors. The Bonferroni-corrected critical *p* value is 05/36 = 0.0014. Values highlighted in bold are statistically significant after Bonferroni corrections for multiple comparisons.﻿

#### Multiple physiological measures

As HR, RR, and SCR, are all responses of the ANS, we assessed which musical features predicted a unified ANS response (i.e., when all three physiological measures were in synchrony simultaneously). Synchrony of the ANS as one entity (HR-RR-SCR) was not possible to model as no LS moments were found in the Dean piece for C1. Splitting ANS responses into paired combinations (HR-RR, HR-SCR, RR-SCR) yielded HS and LS moments in all three styles in all three concerts, allowing for further modelling. Logistic regressions revealed that faster passages (around 120 bpm, see Fig. 2) significantly increased probability of combined RR-SCR synchrony in Brahms C1 and C2 (see Table 4).

**Table 4:** Logistic regressions for combined respiration rate and skin conductance response synchrony per piece across all concerts.

	Beethoven			Dean			Brahms		
	Estimate	SE	*p* value	Estimate	SE	*p* value	Estimate	SE	*p* value
**C1**									
(Intercept)	− 0.880	2.106	0.676	12.646	11.099	0.254	− 6.255	4.129	0.130
Tempo	0.014	0.007	0.039	− 0.036	0.049	0.461	**0.058**	**0.016**	**0.0004**
RMS	82.658	39.948	0.038	535.054	277.231	0.054	− 7.109	36.676	0.8463
S. centroid	0.000	0.001	0.600	− 0.001	0.002	0.508	0.001	0.002	0.323
Key clarity	− 2.644	2.559	0.302	− 18.948	9.346	0.043	− 2.956	3.566	0.407
**C2**									
(Intercept)	0.935	2.486	0.707	− 5.708	7.048	0.418	− 13.149	7.235	0.069
Tempo	0.014	0.006	0.030	0.006	0.034	0.859	**0.211**	**0.067**	**0.002**
RMS	26.386	42.095	0.530	145.701	151.755	0.337	37.250	60.587	0.538
S. centroid	0.000	0.001	0.759	0.002	0.0028	0.549	− 0.001	0.002	0.407
Key clarity	− 4.533	3.077	0.141	3.612	6.376	0.571	− 16.208	9.469	0.087
**C3**									
(Intercept)	− 9.744	3.155	0.002	27.244	22.265	0.221	− 4.988	3.006	0.097
Tempo	0.027	0.011	0.013	0.001	0.0212	0.943	0.025	0.011	0.031
RMS	36.114	37.380	0.334	− 193.756	194.915	0.320	27.009	42.694	0.527
S. centroid	0.002	0.001	0.010	− 0.0146	0.011	0.174	0.000	0.001	0.904
Key clarity	4.609	3.127	0.140	− 11.609	12.505	0.353	2.445	2.863	0.393

Synchrony (HS = 1, LS = 0) was the dependent variable, and tempo, key clarity, loudness, and spectral centroid (s. centroid) from the HS and LS bars as continuous predictors. The Bonferroni-corrected critical *p* value is 0.05/27 = 0.0019. Values highlighted in bold are statistically significant after Bonferroni corrections for multiple comparisons

#### Summary

Although HR, RR, and SCR synchrony were predicted by tempo, RMS, and spectral centroid, the only result that remained consistent across at least two concerts was that synchrony of RR (in Beethoven and Brahms), SCR, and RR-SCR (in Brahms) were predicted by faster tempi. These findings partially support our second hypothesis, showing that one typically arousing music feature (faster tempo) increased the probability of two synchronised arousal-related responses (higher SCR amplitude and increasing RR) consistently, an effect which was modulated by style. Our hypothesis that louder RMS, brighter timbre and lower key clarity would predict physiological synchrony was not supported by the current results.

### Relationship between tempo and subjective experience

As we found a link between faster tempi and the highly synchronised SCR and RR increase (i.e., typical arousal responses) we wanted to further validate whether tempo was indeed related to self-reported emotion and engagement. Therefore, we correlated the mean tempo per movement with the psychological self-report data. Pearson correlations revealed that tempo positively correlated with engagement (e.g., feeling absorbed, concentrated), *r* = 0.475, *p* = 0.003, and positive high arousal emotions (e.g., energetic, joyful), *r* = 0.513, *p* = 0.001, with a medium and strong effect^92^, respectively.

Because the significance of our tempo results varied as a function of musical styles, we explored with descriptive statistics the possibility that familiarity with the piece might play a modulatory role. Beethoven and Brahms pieces were somewhat familiar to over a third of participants (41% and 35%, respectively), while the Dean piece was only somewhat familiar to 2.3% of participants (see Supplementary Table S3), suggesting that one interpretation why tempo significantly predicted physiological synchrony in the Beethoven and Brahms, but not Dean, could be due to differences in familiarity with the music.

### Physiology synchrony across concerts in relation to the music theoretical analysis

With regard to our third hypothesis, we prepared a detailed music theoretical analysis of all the music (see Supplementary Table S4a, S4b, and S4c). We investigated musical events based on this analysis using moments of ‘salient’ physiological synchrony to find time points of interest in the music. Salient physiological responses were operationalised by two criteria: when (1) high physiological synchrony in any of the physiological measures occurred in all three concert audiences and (2) sustained synchrony occurred for more than one bar.

Overall, audience physiology seemed to synchronise around three types of musical events: (a) transitional passages with developing character, (b) clear boundaries between formal sections, and (c) phrase repetitions; all listed with descriptions in Supplementary Tables S5a, S5b, and S5c. Salient responses occurred during calming or arousing transitional passages (*calming*: Beethoven 1st movement, [Beethoven1], bars [b] 85–88; b287–293; Dean2, b70–71; Dean4, b75–77; Brahms4, b75–76; *arousing*: Beethoven1, b303–307; Dean2, b23–25; Brahms3, b6–8), characterised by a decrease (for calming passages) or an increase (for arousal passages) of loudness, texture, and pitch register. Other salient responses occurred when there was a clear boundary between functional sections, indicated through parameters such as a key change (e.g., between major and minor key in Beethoven3, b84–88; Brahms3, b58–61), a tempo change (e.g., Beethoven1 b328–331; Brahms4, b248–250), or a short silence (e.g., Beethoven1, b96–97; Beethoven4, b10–14). Lastly, salient responses occurred when a short phrase or motive was immediately repeated in a varied form, for example in an unexpected key, (e.g., Beethoven1, b35–37), elongated or truncated (e.g., Beethoven1, b85–88; 291–293), or with a different texture or pitch register (Brahms1, b90–91; Brahms3, b170–171). Since the immediate varied repetition of a short phrase is very common in Classical and Romantic styles, salient responses were also evoked when a phrase repetition occurred simultaneously with a transition or clear boundary (e.g., Beethoven1, b24–30; b136–138). With regard to style, the three categories are in line with the compositional conventions of the respective works: salient responses were found more often during transitions in the Romantic and Contemporary works, and during phrase repetitions and boundaries in the Classical work.

## Discussion

This study assessed physiological aspects of the continuous music listening experience in a naturalistic environment using physiological ISC and inter-disciplinary stimulus analyses. We measured physiological responses of audiences listening to live instrumental music performances and examined which musical features evoked systematic physiological responses (i.e., synchronised responses, operationalised via ISC). Consistency of effects was assessed by repeating the same concert three times with different audiences. Importantly, no significant differences of length, loudness, tempo, or timbre across the concert performances were found, allowing us to assume that the musical stimuli were comparable across concerts.

Since previous research has identified typical physiological signatures as indices for felt arousal when listening to music, and since ISC is used to identify reliable responses across several individuals, we firstly hypothesised that ISC would identify similar physiological signatures of arousal. Our results supported this hypothesis: windows of high (compared to low) physiological synchrony contained significantly higher SCR/EMG and increasing RR, depicting similar physiological responses that have been previously related to self-reported arousal/tension evoked by music features like faster tempi^28,32,35,37,38^, peak loudness^61^ as well as unexpected harmonic chords^51,93^ or notes^13^ in music. These patterns of physiological responses indicate that windows of high physiology ISC, therefore, likely correspond to event-related moments of increased arousal.

Our second hypothesis—that low- and medium-level acoustic features can predict high physiological synchrony—was partially supported. Specifically, logistic regressions revealed that one typically arousing musical feature—faster tempi—consistently (i.e., in at least two concerts) predicted synchronised RR and SCR arousal responses. Additionally, tempo consistently predicted a more general ANS response, that is, when both RR and SCR of audience members became synchronised simultaneously. In line with previous work showing that tempo and rhythm are the most important musical features in determining physiological responses^32^, these findings suggest that tempo induces reliable physiological changes.

As faster tempo is typically perceived as more arousing^29–32^, our result that faster music increased probability of high physiological synchrony supports previous research linking high ISC to increased arousal^69,71,82^. Our findings further support the idea that ISC is related to stimulus engagement^9^: as slower music increases mind-wandering^94^, slower tempi may result in reduced attention to the music, leading to greater individual variability in physiological responses and subsequently lower ISC^69,78^. These connections between faster music with high physiological synchrony due to arousal and engagement were further confirmed by the psychological data, where faster tempi were positively correlated with audience members’ self-reported engagement (e.g., high concentration and feelings of being absorbed) and high arousal positive emotions (e.g., feeling energetic or joyful). It is important to note that faster tempi (centred around 120 bpm/2 Hz in the current study) seem to be a more physiologically optimal range for entrainment to music (see^95^ for review). As entrainment is difficult at note rates under 1 Hz (at least for non-expert musicians^96^), this may explain why synchrony did not occur in slower tempi of the current musical stimuli (which was centred around 50 bpm/0.83 Hz). It is therefore reasonable to speculate that entrainment, or the adaptation of autonomic physiological measures towards the musical tempo, might be a mechanism through which faster or optimally resonant tempi induce more-similar audience responses. In summary, we postulate that physiological synchrony may be predicted by faster music because of the physiological properties of the nervous system, which, when optimally driven, induce specific psychological experiences such as increased arousal and engagement.

It is of further interest that SCR and RR synchrony were more probable at faster tempi in the Classical and Romantic styles, but not in the Contemporary style, supporting previous studies where the same features evoke different physiological responses based on the style^34,62^. As Beethoven and Brahms were rated as more familiar compared to Dean, ISC differences between styles in the current results support a recent study showing that ISC is linked to familiarity with musical style^9^. Additionally, Beethoven and Brahms have relatively stable meters (i.e., very few instantaneous tempo changes within movements), whereas many Dean passages contained unpredictable meters (e.g., the first movement has alternating bars of four, five, or six beats per bar) and frequent tempo changes within movements. In view of this, the fact that synchrony probability changes between styles could be due to stimulus coherence, corroborating studies showing that higher ISC occurs in more predictable contexts^78^ and lower ISC occurs in versions of music where the beat is disrupted^80^. However, since we presented only one work per style (and tempo changes were not evenly represented across these styles)—a compromise dictated by the constraints of a naturalistic concert setting, complimentary research using a wider range of pieces (in different styles) is required to further assess the effects of coherence and familiarity on synchrony.

Although we show that synchrony was predicted by tempo (depending on the style), the hypothesis that louder RMS, brighter timbre, and lower key clarity would predict physiological synchrony was not met. This was unexpected, as orienting/startle response research consistently shows that loudness evokes highly replicable physiological responses in a controlled tone sequence^24,27,28^. Because loudness in the current study was embedded in naturalistic music, our findings highlight the generalisability limitations of reductionist stimuli to real-world contexts^1^. Indeed, previous work has shown that environmental sounds and music evoke different physiological responses; an increase in HR (index of a startle response^23^) occurred with arousing noises (e.g., a ringing telephone or storm), but not with music^97^. However, as we used such naturalistic music, it was important to not only explore quantitatively extracted low- and medium-level features, but also higher-level parameters in the music.

With regard to the hypothesis that synchrony corresponds to high-level/structural moments in the music, we observed that (from all moments in the music) transitional passages, clear boundaries, and immediate phrase repetitions in the music—identified using music theoretical analysis—coincided with highly synchronised physiological responses across concerts. Similar high-level features have been associated with physical responses such as shivers, laughter or tears (see Table IV in ^54^) or specifically chills^61^, which also typically correspond with increased physiological arousal^55–60^. However, none of the previous studies have derived these categories based on a detailed analysis of complete pieces and in the context of longer time spans.

In the current study, synchronised physiological responses occurred during arousing/calming transitional passages (characterised by changes in loudness, pitch register, and musical texture) and boundaries indicated by sudden tempo or key changes. Previous research has shown that unexpected musical events embedded in a predictable context may be perceived as arousing^13,51,93^, further corroborating findings that high ISC occurs at arousing moments^69,71,82^. Our results additionally align with the notion that audience members collectively ‘grip on’ to loudness and texture changes^5^. The finding that physiological synchrony occurred during tempo changes, supports the fact that disruptions of temporal expectations affect ANS responses^28,98^ and EEG synchrony^80^, where surprising events phase-reset ongoing physiological oscillations (see^99,100^ for reviews), leading—at least briefly—to an increase in audience synchrony around moments of phase resetting.

We also found that momentary synchronised responses occurred during short phrases which were immediately repeated in a varied form, hinting at a general attention towards repetitions in music^101^ and a recognition of thematic connections over longer time spans^101^. Our analysis further suggests that an interplay of various musical features—in addition to the simple repetition—increase attention, and subsequently synchrony, of all audience members to these musical moments. For instance, high audience synchrony occurred in some of the structurally most important moments of Beethoven’s 1st movement, where phrase repetition occurs simultaneously with a boundary (at the end of the exposition and with references to the primary theme at the end of the movement), and a boundary occurs simultaneously with transitional passages (deferred cadences; declined structural closures: e.g., b96–97, b301–302). The fact that high ISC occurred at phrase repetitions with an added novelty in the phrase (e.g., in a different key)—and also at important structural locations—not only supports research where novelty in stimuli evokes a physiological response^16,18,21–28^, but is also in line with music compositional practices, in which a composer tries to vary and develop thematic material^102^ (with different textures and/or harmonies) to keep listeners’ interest.

In conclusion, by measuring continuous music listening experience in a naturalistic setting of a chamber music concert, we show that systematic synchronised physiological responses (corresponding to typical arousal responses) across audience members are predicted by tempo (depending on style) and are linked to structural transitions, boundaries, and phrase repetitions. Using naturalistic music in a concert environment is beneficial in that participants are likely to have more realistic and stronger responses^2–4^. However, as this benefit makes our findings specific to the music we have used, future research should assess whether the current findings related to musical features and style are replicated with different (styles of) music. Additionally, further questions remain regarding the concert setting itself; for example, whether physiological effects and subjective experiences change with/without visual movements of the performer(s), with varying programming orders, and in different performance spaces^6,103^. Exploring musical experiences from pre-recorded or live performances—with or without the co-presence of others—may prove an interesting future research direction, especially with regard to the COVID-19 pandemic and current transformations of the concert itself.

## Method

### Experimental procedures

Experimental procedures are identical to Merrill et al.^104^. All experimental procedures were approved by the Ethics Council of the Max Planck Society, and undertaken with written informed consent of each participant. All research was performed in accordance with the Declaration of Helsinki.

### Participants

129 participants attended one of three evening concerts. Some data were lost due to technical issues of server and user failures (*N* = 31) during data acquisition, leaving data from 98 participants for analysis. Gender and age were similarly distributed across concerts (see Table 5). Most participants reported that their highest level of education was a university degree, a German high school degree, or completed professional training. Musical Sophistication—assessed with the General Music Sophistication and Emotions subscales from the German version of the Goldsmiths Music Sophistication Index^105,106^—was similarly moderate (see Appendix Table 3 in^101^) across the three audience groups (see Table 5). Most participants reported that they regularly attend classical concerts and opera.

**Table 5:** Demographic information about participants in the current study, showing distribution of age, gender, and musical sophistication (general and emotions) across concerts.

Concert	Total *N*	Gender	Age	Gold-MSI: emotion	Gold-MSI: general
				Mean score (SD)	Mean score (SD)
C1	36	F = 15, M = 17, *na* = 4	50% < 50 years old	33.53 (4.75)	69.84 (22.01)
C2	41	F = 16, M = 17, *na* = 8	50% < 55 years old	31.17 (6.85)	71.61 (21.97)
C3	21	F = 9, M = 12	50% < 40 years old	33.24 (5.45)	70.76 (19.33)

Participants were asked to report their age by selecting age group (within a 5-year range from 18 to 99, i.e., 18–22, 23–27, 28–32, etc.).

### Concert context

Three evening concerts (starting at 19.30 and ending at approximately 21.45) took place in a hybrid performance hall purpose-built for empirical investigations (the ‘ArtLab’ of the Max Planck Institute for Empirical Aesthetics in Frankfurt am Main, Germany). Care was taken to keep parameters (e.g., timing, lighting, temperature) as similar as possible across concerts. Professional musicians performed string quintets in the following order: Ludwig van Beethoven, op. 104 in C minor (1817), Brett Dean, ‘Epitaphs’ (2010), and Johannes Brahms, op. 111 in G major (1890), with a 20-minute interval between Dean and Brahms. This program was chosen to represent a typical chamber music concert.

### Procedure

Participants were invited to arrive either one or one and half hour(s) before the concert began for physiological measurement preparation. Physiology was measured with a portable recording and amplifying device (https://plux.info/12-biosignalsplux) for the whole concert at 1000 Hz. Continuous blood volume pulse (BVP) was measured using a plethysmograph clip; respiration data were measured using a respiration belt (wrapped snugly around the lower rib cage); skin conductance was measured with electrodes placed on index and middle fingers of the non-dominant hand; and facial muscle activity was measured using electromyography, with adhesive electrodes placed above the zygomaticus major (‘smiling’) muscle on the left side of the face and ground placed on the mastoid. After each of the 12 movements, a short (2-min) pause was taken for the participants to fill in two questionnaires. The first questionnaire measured state absorption: eight items (e.g., ‘I was completely absorbed by the music’, ‘My mind was wandering’) were rated on a 5-point Likert scale from ‘strongly disagree’ to ‘strongly agree’^107^. The second questionnaire measured intensity of felt emotions using the **GE**neva **M**usic-**I**nduced **A**ffect **C**hecklist (GEMIAC^108^), where intensity of 14 classes of feelings (e.g., energetic/lively, tense/uneasy, nostalgic/sentimental) were rated on a 5-point Likert scale from ‘not at all’ to ‘very much’ (intensely experienced). Items were presented in German using comparable adjectives from the German version of the AESTHEMOS^109^. After each piece, participants rated how familiar the piece was (‘Yes’, ‘No’, and ‘Not sure’).

### Data analysis

#### Musical feature extraction

Instantaneous tempo was calculated using inter-onset intervals (IOIs) between each beat (where beats were manually annotated by tapping each beat using Sonic Visualiser^102^). These IOIs were then converted to beats per minute (bpm). All other features were computationally extracted using the MIRToolbox (version 1.7.2)^110^ in MATLAB 2018b. In order to capture musical features meaningfully, different time windows were used to extract certain musical features. For features that change quickly on a timeframe of a less than a second—i.e., RMS energy (related to loudness), spectral centroid, brightness, and roughness (related to timbre)—a time window of 25 ms with a 50% overlap was used, as is typical in the music information retrieval literature^8,111^. Other features that are more context-dependent (that develop over a longer time frame), such as key clarity (i.e., the reverse of harmonic ambiguity) require a longer time-window, and were extracted similar to previous studies that assess the same musical feature, that is using a 3 s window^8,41^ with 33% hop factor (overlap)^112^. As previous time-series analyses have parsed data into meaningful units of clause and sentence lengths^74^, and as we wanted to aligned responses across concerts, each feature was averaged into a meaningful and comparable music unit: a bar (American: measure, on average 2 s long). It is worth noting that acoustic features can be distinguished between compositional features and performance features^113^, where the former are represented in the musical score (such as harmony) while the latter include features that can change between performances, namely how loud and fast musicians may perform the music. Because key clarity is a compositional feature (i.e., does not change between performances), we had the same values across all three concert performances.

When checking for independence of features^114^, Pearson correlations revealed that RMS and roughness correlated highly as did brightness and spectral centroid (all *r* > 0.7, *p* > 0.0001) in all movements. As RMS and spectral centroid are features more commonly used, compared to roughness and brightness^113,115^, and spectral centroid seems to best represent timbre^83,116^ and brightness^117^ perception, we kept only key clarity, RMS, spectral centroid, and tempo for further analysis. To check performance feature similarity between concerts, features were compared with concert (C1, C2, C3) as the independent variable. Pearson correlations were used to assess similarity of acoustic features over time between concerts. Correlations were considered adequate if they met a large effect size of concert *r* > 0.5^92^. To compare acoustic features per style, linear mixed models with fixed effect of the works (Beethoven, Brahms, and Dean) and random intercept of movement were constructed with each acoustic feature (as the dependent variables) per concert.

#### Physiology pre-processing

Physiological data were pre-processed and analysed in MATLAB 2018b. Data were cut per movement. Missing data (gaps of less than 50 ms) were interpolated at the original sampling rate. Fieldtrip^118^ was used to pre-process BVP, respiration, and EMG data. BVP data were band-pass filtered between 0.8 and 20 Hz (4th order, Butterworth) and demeaned per movement. Adjacent systolic peaks were detected to obtain inter-beat intervals (IBIs) and an additional filter was added to remove any IBIs that were shorter than 300 ms, longer than 2 s, or had a change of more than 20% between adjacent IBIs (typical features of incorrectly identified IBIs^119^). After visual inspection and artefact removal, IBIs were converted to continuous heart rate (HR) by interpolation. Respiration data were low-pass filtered (0.6 Hz, 6th order, Butterworth) and demeaned. Maximum peaks were located and respiration rate (RR) was inferred by the peak intervals. EMG activity was band-pass filtered (between 90 and 130 Hz, 4th order, Butterworth), demeaned, and the absolute value of the Hilbert transform of the filtered signal was extracted and smoothed. Skin conductance data were pre-processed using Ledalab^15^ and decomposed into phasic and tonic activity. As we were interested in event-related responses, only (phasic) skin conductance responses (SCR) were used in further analyses. All pre-processed physiological data (SCR, HR, RR, EMG) were resampled at 20Hz^17^, *z*-scored within participant and movement, and averaged into bins per bar.

#### ISC analysis

We calculated a time-series ISC based on Simony et al.^78^ by forming *p* × *n* matrices (one for each SCR, HR, RR, and EMG, and for each of the twelve movements per concert), where *p* is the physiological response for each participant over *n* time points (bars across the movements). Correlations were calculated over a sliding window 5-bars long (approximately 10 s; the average bar length across the whole concert was 2 s), shifting one bar at a time. Fisher’s *r*-to-*z* transformation was applied to correlation coefficients per subject, then averaged *z* values were inverse transformed back to *r* values. The first 5 bars and the last 5 bars of each movement were discarded to remove common physiological responses evoked by the onset/offset of music^76^. ISC values per movement were concatenated within each concert (2238 bars), giving four physiological ISC measures per concert. These ISC traces represent the similarity of the audience members’ physiological responses over time (see Fig. 1a).

Following Dmochowski et al.^71^, high and low synchrony were defined using 20th percentiles. Windows containing the highest 20th percentile of ISC values were categorised as high synchrony (HS) windows. Windows with values in the lowest 20th percentile of correlation values (i.e., ISC values within a 20th percentile where *r* was centred around zero) were categorised as low synchrony (LS) windows. To obtain instances of overall ANS synchrony (i.e., across multiple physiological measures simultaneously), we identified where HS/LS moments of one physiological measure coincided with another physiological measure. Physiological responses at points of HS and LS were compared using linear models with fixed effects Synchrony (high/low) and Bar (bars 0–4). To investigate whether acoustic features predicted synchrony (HS/LS) of physiological responses across audience members, tempo, RMS energy, key clarity, and spectral centroid in bars of HS/LS were recovered. By dummy-coding Synchrony as a binary variable (HS as 1; LS as 0) logistic regression models were constructed to predict Synchrony for each physiological measure (dependent variable) with continuous predictors of tempo, key clarity, loudness, and spectral centroid from the HS and LS bars (all features were included, as perceived expression in music tends to be determined by multiple musical features^30^). (N.B.: no random intercept of movement was included, because not all movements contained HS/LS epochs). As we expected style to modulate the effect of these acoustic features in predicting synchrony, models were run separately per piece and concert.

#### Subjective ratings

A factor analysis was conducted for all ratings from the absorption and GEMIAC scales. Five latent variables were identified: (1) Positive high arousal emotions (energetic, powerful, inspired, joyful, filled with wonder, enchanted); (2) Negative high arousal emotions (tense, agitated, negative loadings of liking and feeling relaxed); (3) Mixed valence, low arousal emotions (relaxed, nostalgic, melancholic, feelings of tenderness, moved); (4) Engagement (concentrated, forgetting, absorbed, liking, with negative loadings of bored, indifferent, and mind-wandering); and (5) Dissociation (forgetting being in a concert and forgetting surroundings); see^104^ for their exact loadings. We (Pearson) correlated participants’ factor scores with musical features.

#### Music theoretical analysis

The scores of all works were analysed according to widely used methods for the respective styles. Musical events were analysed on the beat level (harmonic changes, cadences, texture changes, motivic relation^120^) and grouped into larger sections (thematic relations, formal functions/action spaces, repetition schemata^89,90^). The performance recordings served as references for passages which could have been interpreted equivocally in the score. After the analysis, passages involving high physiological synchrony were marked. These musical features were compared with each other, categorized across styles, and finally reduced to three categories: (a) transitional passages, (b) clear boundaries between formal sections, and c) phrase repetitions.

#### Statistical analyses

Statistical analyses were conducted in R^121^. Pearson correlations were computed using *corr.test* in the *psych* package^122^ and adjusted for false discovery rate using the Benjamin-Hochberg procedure. Linear (mixed) models were constructed using the *lme4* package^123^; *p* values were calculated with the *lmerTest* package^124^ and using the *Anova* function in the *car* package^125^. Contrasts were assessed with the emmeans function (*emmeans* package^126^). Logistic regression models were run using a general linear model with a logit link function. Significance thresholds for *p* values for ANOVA, contrasts, and logistic regression models were adjusted using Bonferroni corrections.

## Supplementary Information

Supplementary Information.

## Data Availability

Data of this study are available from the corresponding author upon request.
