# Supplementary material for: Synchrony in the periphery: inter-subject correlation of physiological responses during live music concerts
Source: Sci Rep. 2021 Nov 17;11:22457. doi: 10.1038/s41598-021-00492-3 (PMC8599424; doi:10.1038/s41598-021-00492-3)
Supplement: Supplementary file 1 — Supplementary Information. [file 41598_2021_492_MOESM1_ESM.pdf]

Synchrony in the periphery: inter-subject correlation of physiological responses during live music concerts

Anna Czepiel[1]*, Lauren K. Fink[1,2], Lea T. Fink[1], Melanie Wald-Fuhrmann[1,2], Martin Tröndle[3], Julia Merrill[1,4]

[1] Max Planck Institute for Empirical Aesthetics, Frankfurt am Main, Germany

[2] Max Planck - NYU Center for Language, Music, & Emotion (CLaME), New York, USA

[3] Zeppelin University, Friedrichshafen, Germany

[4] Institute of Music, University of Kassel, Kassel, Germany

Supplementary Information

Supplementary Table S1a. ANOVA for performance (acoustic) differences across concerts. ANOVA was calculated using Anova function in car package in R.

		Beethoven		Brahms		Dean	
	df	F	*p*	F	*p*	F	*p*
RMS	2	2.14	0.12	0.23	0.79	0.12	0.89
Spectral centroid	2	1.78	0.17	1.54	0.21	2.82	0.06
Tempo	2	2.56	0.08	0.02	0.98	2.31	0.10

Supplementary Table S1b. Lengths of separate movements of works.

	Beethoven movements				Dean movements				Brahms movements			
	1st	2nd	3rd	4th	1st	2nd	3rd	4th	1st	2nd	3rd	4th
C1	7.80	8.15	3.76	6.37	2.53	3.87	3.90	3.39	10.17	7.15	5.37	4.94
C2	7.95	8.36	3.74	6.50	2.70	3.87	3.93	3.49	10.24	7.07	5.39	4.95
C3	7.75	8.02	3.71	6.23	2.68	3.87	3.79	3.14	10.03	6.97	5.46	4.93

Supplementary Table S1c. Correlations between features within movements. Bonferroni corrected *p* value was *p* = .0003. All correlations significance values we still under this threshold and, therefore, significant. Correlations were run using *corr.test* function from the *psych* package in R.

	RMS			Spectral Centroid			Tempo		
	Comparison	*r*	*p*	Comparison	*r*	*p*	Comparison	*r*	*p*
Bee1	C1 - C2	.947	> .0001	C1 - C2	.8760	> .0001	C1 - C2	.822	> .0001
	C1 - C3	.946	> .0001	C1 - C3	.889	> .0001	C1 - C3	.830	> .0001
	C2 - C3	.957	> .0001	C2 - C3	.927	> .0001	C2 - C3	.841	> .0001
Bee2	C1 - C2	.875	> .0001	C1 - C2	.901	> .0001	C1 - C2	.858	> .0001
	C1 - C3	.855	> .0001	C1 - C3	.908	> .0001	C1 - C3	.861	> .0001
	C2 - C3	.839	> .0001	C2 - C3	.900	> .0001	C2 - C3	.832	> .0001
Bee3	C1 - C2	.887	> .0001	C1 - C2	.940	> .0001	C1 - C2	.811	> .0001
	C1 - C3	.884	> .0001	C1 - C3	.910	> .0001	C1 - C3	.821	> .0001
	C2 - C3	.916	> .0001	C2 - C3	.882	> .0001	C2 - C3	.824	> .0001
Bee4	C1 - C2	.922	> .0001	C1 - C2	.920	> .0001	C1 - C2	.684	> .0001
	C1 - C3	.904	> .0001	C1 - C3	.906	> .0001	C1 - C3	.628	> .0001
	C2 - C3	.902	> .0001	C2 - C3	.888	> .0001	C2 - C3	.622	> .0001
Bra1	C1 - C2	.964	> .0001	C1 - C2	.978	> .0001	C1 - C2	.848	> .0001
	C1 - C3	.947	> .0001	C1 - C3	.962	> .0001	C1 - C3	.844	> .0001
	C2 - C3	.956	> .0001	C2 - C3	.973	> .0001	C2 - C3	.881	> .0001
Bra2	C1 - C2	.975	> .0001	C1 - C2	.965	> .0001	C1 - C2	.893	> .0001
	C1 - C3	.974	> .0001	C1 - C3	.961	> .0001	C1 - C3	.871	> .0001
	C2 - C3	.984	> .0001	C2 - C3	.977	> .0001	C2 - C3	.932	> .0001
Bra3	C1 - C2	.957	> .0001	C1 - C2	.933	> .0001	C1 - C2	.595	> .0001
	C1 - C3	.940	> .0001	C1 - C3	.939	> .0001	C1 - C3	.692	> .0001
	C2 - C3	.950	> .0001	C2 - C3	.932	> .0001	C2 - C3	.645	> .0001
Bra4	C1 - C2	.957	> .0001	C1 - C2	.936	> .0001	C1 - C2	.843	> .0001
	C1 - C3	.961	> .0001	C1 - C3	.920	> .0001	C1 - C3	.831	> .0001
	C2 - C3	.952	> .0001	C2 - C3	.931	> .0001	C2 - C3	.848	> .0001
Dea1	C1 - C2	.848	> .0001	C1 - C2	.751	> .0001	C1 - C2	.987	> .0001
	C1 - C3	.876	> .0001	C1 - C3	.843	> .0001	C1 - C3	.992	> .0001
	C2 - C3	.939	> .0001	C2 - C3	.895	> .0001	C2 - C3	.991	> .0001
Dea2	C1 - C2	.999	> .0001	C1 - C2	.982	> .0001	C1 - C2	1	> .0001
	C1 - C3	.998	> .0001	C1 - C3	.976	> .0001	C1 - C3	1	> .0001
	C2 - C3	.998	> .0001	C2 - C3	.990	> .0001	C2 - C3	1	> .0001
Dea3	C1 - C2	.982	> .0001	C1 - C2	.972	> .0001	C1 - C2	.940	> .0001
	C1 - C3	.971	> .0001	C1 - C3	.955	> .0001	C1 - C3	.858	> .0001
	C2 - C3	.975	> .0001	C2 - C3	.944	> .0001	C2 - C3	.913	> .0001
Dea4	C1 - C2	.947	> .0001	C1 - C2	.936	> .0001	C1 - C2	.936	> .0001
	C1 - C3	.944	> .0001	C1 - C3	.931	> .0001	C1 - C3	.891	> .0001
	C2 - C3	.940	> .0001	C2 - C3	.936	> .0001	C2 - C3	.887	> .0001

Note. Dean 2 was played via a recording, hence the perfect correlation for tempo across concerts (and near perfect for loudness and spectral centroid, with slight possible deviations in the audio recording).

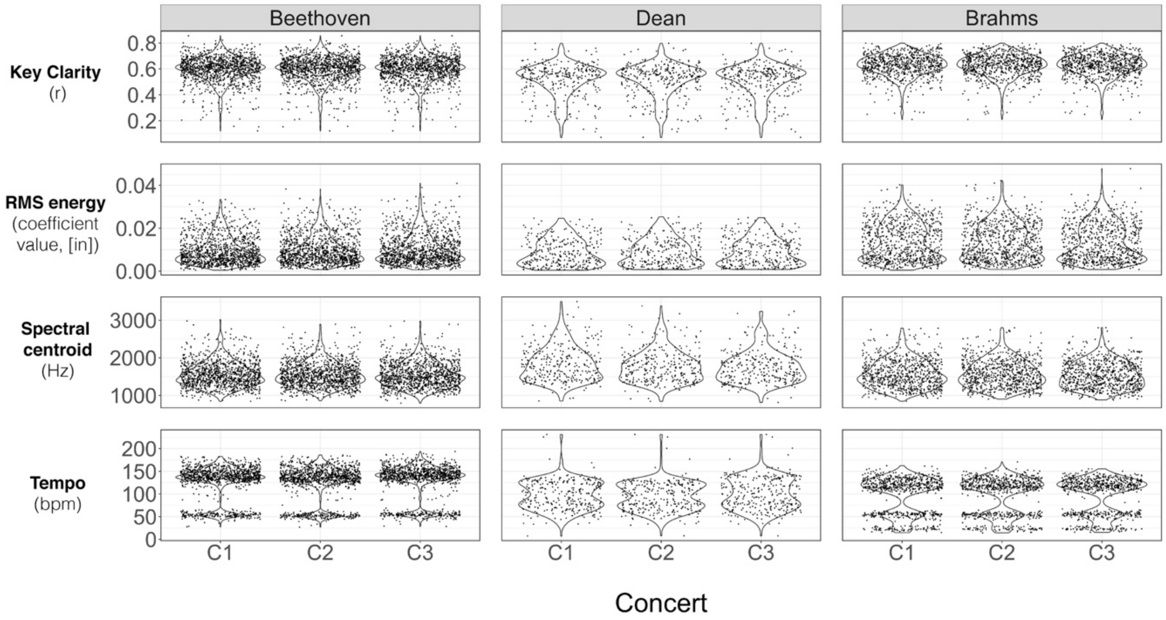

Supplementary Figure S1. Acoustic features per bar, per piece, per concert. Top to bottom panels show Key clarity, RMS energy, Spectral centroid, and tempo values per bar. Left panels show values for Ludwig van Beethoven (String Quintet in C minor, op. 104, 1817), middle panels for Brett Dean (Epitaphs, 2010), and right panels for Johannes Brahms (String Quintet in G major, op. 111, 1890). Separate violin plots show different concerts.

Supplementary Table S2a. Musical feature differences between styles. ANOVA was calculated using *Anova* function in *car* package in R. Values highlighted in bold are statistically significant.

		C1		C2		C3	
Acoustic feature	df	χ^2	*p*	χ^2	*p*	χ^2	*p*
RMS	**2**	**9.832**	**.007**	**9.853**	**.007**	**8.788**	**.012**
Spectral centroid	**2**	**21.630**	**< .001**	**12.165**	**.002**	**13.736**	**.001**
Key clarity	**2**	**28.738**	**> .001**	**28.738**	**> .001**	**28.738**	**> .001**
Tempo	2	2.136	.343	2.125	.346	2.237	.327

Note. As key clarity was the same across all concerts, there was no change in the difference between pieces between concerts.

Supplementary Table S2b. Pairwise comparisons of models comparing acoustic features between different pieces. Contrasts (Bonferroni adjusted) were calculated with *emmeans* package in R. Values highlighted in bold are statistically significant.

Feature	Concert	Pairwise comparison	Estimate	SE	df	*t*	*p*
RMS	C1	Beethoven-Brahms	-0.004	0.002	8.46	-2.423	.120
		Beethoven– Dean	0.001	0.002	9.22	0.571	1.00
		**Brahms – Dean**	**0.005**	**0.002**	**9.42**	**2.923**	**.049**
	C2	Beethoven-Brahms	-0.004	0.002	8.40	-2.361	.101
		Beethoven– Dean	0.001	0.002	9.25	0.676	1.00
		**Brahms – Dean**	**0.005**	**0.002**	**9.48**	**2.959**	**.045**
	C3	Beethoven-Brahms	-0.003	0.002	8.40	-2.090	.205
		Beethoven– Dean	0.001	0.002	9.25	0.836	1.00
		**Brahms – Dean**	**0.005**	**0.002**	**9.48**	**2.856**	**.054**
Spectral centroid	C1	Beethoven-Brahms	0.54	76.6	8.39	0.007	1.00
		**Beethoven– Dean**	**-320.36**	**78.7**	**9.26**	**-4.072**	**.008**
		**Brahms – Dean**	**-320.90**	**79.2**	**9.49**	**-4.053**	**.008**
	C2	Beethoven-Brahms	-12.2	87.3	8.54	-0.139	1.00
		**Beethoven– Dean**	**-277.0**	**89.0**	**9.18**	**-3.112**	**.037**
		**Brahms – Dean**	**-264.**	**89.4**	**9.35**	**-2.962**	**.046**
	C3	Beethoven-Brahms	-0.828	75.8	8.42	-0.011	1.00
		**Beethoven– Dean**	**-252.462**	**77.7**	**9.24**	**-3.251**	**.029**
		**Brahms – Dean**	**-251.633**	**78.1**	**9.45**	**-3.221**	**.030**
Key clarity		Beethoven-Brahms	-0.031	0.021	8.40	-1.495	.541
		**Beethoven– Dean**	**0.080**	**0.021**	**9.25**	**3.791**	**.012**
		**Brahms – Dean**	**0.111**	**0.021**	**9.48**	**5.216**	**.001**

Note. As key clarity was the same across all concerts, there was no change in the difference between pieces between concerts.

Supplementary Table 3. Familiarity ratings across concerts that were collected after each piece to the question if participants knew the piece (with three possible choices: yes, no, not sure).

	Familiar	Not sure if familiar	Unfamiliar
Beethoven	12.5%	28.4%	59.1%
Brahms	15.9%	19.3%	65.9%
Dean	0%	2.3%	97.7%

Ludwig van Beethoven: Quintet op. 104, c minor. Music theoretical analysis of Mvt. 1, Allegro con brio, bars 1-137 (exposition)

Supplementary Table S4a: Complete movement, division of formal sections

from bar	to bar	form part	length in bars
1	30	Exposition - Primary zone	*30*
31	58	Exposition - Transition	*28*
59	124	Exposition - Secondary zone	*66*
124	137	Exposition - Closing zone	*14*
138	213	Development zone	*76*
214	223	Recapitulation - Primary Zone	*10*
224	261	Recapitulation - Transition	*38*
262	327	Recapitulation - Secondary Zone	*66*
328	360	Recapitulation - Closing Zone	*33*

Supplementary Table S4b: Sample analysis of the exposition with broad function, harmonic context / development, phrase structure and dynamic relations

from bar	to bar	event bar.beat	function	phrase structure	harmonic context	dynamics (descriptive)
1	30		Exposition - Primary zone		i = c minor	
1	10		P^{0}	4+2+2+2	c minor	soft
		3-4	basic idea: arpeggiated V (sixth chord)			
		5-6	b.i. repetition, on VI (sixth chord)		A♭ major	
		9.1	delay, ornamented cadence			soft
		9.2	ornament: highpoint of the melody, seventh			
		9.3	rhethoric delay			
		10.1	HC, 9-8 suspension, fermata / delay		G major	soft
11	14		$P^{\text{pres. 1}}$: compound basic idea	2+2	c minor	soft
		13-14	HC, 4-3 suspension			
15	18		$P^{\text{pres. 2}}$: prolinging the tonic	2+2		soft
		17-18	IAC, 4-3 suspension			soft
19	30		$P^{\text{cont.}}$, pedal point on dominant, pendulum V-i (D-t), pre-cadence	6+2+2+2		increasing
		19.1	start of pedal point			accent
		21	increasing density, syncopations on 19.2, 20.2, 21.2 etc.			increasing
		27.1	dynamic maximum			strong
		27.3	dynamic maximum			strong
		28.1	dynamic maximum			strong
		29-30	HC		G major	strong
31	58		Exposition - Transition		i --> III	
31	39		TR-P^{0}, transposed, shortened	4+2+2	A♭ major	soft
		31.1	harmonicaly surprising shift to VI (tG)			strong
		33-34	compare bars 3-4, motive: arpeggiated V (sixth chord)			
		35-36	compare bars 5-6, motive repetition, on VI (sixth chord)		F♭ major	
		37.1	ante penultima - compare to bar 7			strong
		38.1	penultima, leadington in the bass			strong
37	39		chromatic bassline progression to B♭, dominant of III (D)tP	2+1		increasing
39	57		"standing on the dominant", structural pedal point on B♭		B♭ major	
40	43		TR-$P^{1\ \text{ant}}$	2+2	(V-i)	increasing
44	47		TR-$P^{1\ \text{cons}}$	4+4		increasing
47	53		TR-precadence, Takterstickung	4+3	(V-i)	strong
		53.1	MC declined			strong
53	56		cadence, falling bassline	4+2		decreasing
57	58		Takterstickung, MC-fill			
		57.1	MC deformed: HC V of III (tP)		B♭ major	
59	124		Exposition - Secondary zone		I = E♭ major	
59	66		$S^{1.1}$, antecedent+consequent	4+4	E♭ major	soft
67	75		$S^{1.2}$, transposed repetition of S^{1}, modulation to IV	4+4+1		soft
		69.1	beginning: new tonal region: IV		A♭ major	soft
		75.1	IV: IAC			
		75	sustaining transitional bar			
76	79		$S^{2\text{ant}}$	2+2		decreasing
		79.1	IV: HC			
80	91		$S^{2\text{con}}$, pedalpoint on E♭, modulation to V dimin (1-->7th)	2+2+2+2+1+1+1+1	e♭ - a♭ - Fdim.	decreasing
		91.1	accent, syncopated phrase rhythm			strong
92	97		pre-cadence V, harmonic rhythm accelerated	4+2	B♭ major	strong
		96-97	V: HC, 64-53 suspension			
		98.1	I: evaded PAC, declined EEC		E♭ major	strong
98	109		sequences of P^{0}, pre-cadential	4+2+2+1+1+1+1		strong
110	118		$S^{3.1}$, augmentation of ornamenting neighbournotes, IAC	4+4		
		111.1	Neopolitan chord		F♭ major	
118	124		$S^{3.2}$, augmentation of ornamenting neighbournotes, IAC	3+3		arching
		118.1	Deceptive cadence		C♭ major	
124	137		Exposition - Closing zone	4+4	I = E♭ major	increasing
		124.1	PAC, EEC		E♭ major	strong
132	137		cadence	2+2+2		strong

Supplementary Table S4c: Terminology & References

Material:	P, S, TR = Primary theme, Secondary theme, Transition (Hepokoski 2006)
Temporal labels:	$P^{0,\ 1,\ \text{etc.}}$ = Primary Theme and its consecutive sections (0 as introduction, 1 as first part of theme one, etc.), e.g. TR-P^{0} = section of transition with material that originates in the primary zone (Hepokoski 2006)
Phrase structure:	ant. / cons. / pres. / cont. = antecedent / consequent / presentation / continuation. (Caplin 1998)
Cadences:	HC = half cadence, IAC = imperfect authentic cadence, PAC = perfect authentic cadence, DC = deceptive cadence
Functional cadences:	EEC = essential expositional closure, ESC = essential structural closure, MC = medial caesura (Hepokoski 2006)

Caplin, W. E. (1998). *Classical Form : A Theory of Formal Functions for the Instrumental Music of Haydn, Mozart, and Beethoven.* New York: Oxford University Press

Hepokoski, J. A., & Darcy, W. (2006). *Elements of sonata theory: Norms, Types, and Deformations in the Late Eighteenth-Century Sonata* . New York: Oxford University Press.

Supplementary Table S5a. Musical descriptions of bars with salient physiological responses: Beethoven

Ludwig van Beethoven: String Quintet in C minor op. 104 (1817)			
**Movement**	**Bars**	**Categories**	**Description**
1st *	24-30	Phrase repetition<br>Transition	Exposition: Increasing texture, dynamics & harmonic rhythm, modulation and half-cadence bar30 ending the primary-theme zone (P)
	35-37	Phrase repetition	Exposition: Chromatic sequence at opening of transition (T)
	85-88	Phrase repetition<br>Transition	Exposition: Decreasing texture & dynamics, bar-wise repetition of motif, elongation & prolongation of secondary diminished dominant
	96-97	Boundary	Exposition: Strong half-cadence, essential expositional closure (EEC) declined in b97
	136-138	Phrase repetition<br>Boundary	Exposition: Increasing texture & dynamics, syncopations, cadence ending the closing zone (C) of exposition. First chord of development section.
	287-290	Phrase repetition<br>Transition	Recapitulation: Decreasing texture & dynamics, bar-wise repetition of motif, prolongation of secondary diminished dominant
	291-293	Phrase repetition	Recapitulation: Elongation & prolongation of secondary diminished dominant
	301-302	Boundary	Recapitulation: essential structural closure (ESC) declined in b300, beginning of strong transitional section, motivic development of P in unison
	303-307	Transition	Recapitulation: strong transitional section, motivic development of P in unison
	328-331	Boundary	Coda: Reference to opening bars with fermata & tempo change (150bpm to Adagio, ca. 80 bpm and back) after final cadence in b327
2nd	62-64	Boundary	Variation 1: Perfect authentic cadence (PAC), end of var 1
	126-130	Transition<br>Boundary	Variation 3: increase of dynamic (*morendo*), PAC b129 ending var. 3 in minor.<br>Variation 4: Key, texture, tempo change, beginning of var.4 in major
3rd	65-67**	Phrase repetition	Menuetto 1, B-part 2nd time: standing on the dominant after half cadence (HC) b64, reference to beginning of B-part b55-58
	84-88	Boundary	Menuetto 1, A'-part 2nd time: end of menuetto (minor), beginning of Trio (major). Change of key, register, texture, phrase rhythm
4th *	10-14	Boundary<br>Transition	Exposition: beginning of primary theme one (P1) after intro & general rest in b8
	150-153	Phrase repetition<br>Boundary	Development: continuation within P0 at the beginning of development,<br>half cadence, general rest
	187-188	Phrase repetition<br>Boundary	Development: rotation of subordinate theme (S), second pair of of six-bar chromatic sequence of S

**Note*: no repeat. ***Note*: Bars in relation to repeats

Supplementary Table S5b. Musical descriptions of bars with salient physiological responses: Dean

Brett Dean: Epitaphs (2010)			
**Movement**	**Bars**	**Categories**	**Description**
2nd	23-25	Transition	Increasing texture density, rhythmically clearer than previous context
	61-62	Transition	Closing phrase with decreasing loudness
		Boundary	cadence and *finalis* with fermata
	70-71	Transition	Increasing loudness and dissonance, varied repetition, change of pitch class quality & timbre (*increasingly raw*)
4th	75-77	Boundary	Sudden decrease of dynamics, change of texture & timbre
		Transition	Glissando over 3+3+2 pattern

Supplementary Table S5c. Musical descriptions of bars with salient physiological responses: Brahms

Johannes Brahms: String Quintet in G major op. 111 (1890)			
**Movement**	**Bars**	**Categories**	**Description**
1st *	90-91	Phrase repetition	Development: combined P/S space, homophonic texture
3rd	6-8	Transition	1st minor part, A: modulation to half cadence in the middle of 8-bar continuation. Small increase of dynamics, descending line in upper melody
	58-61**	Boundary	1st minor part, B: end of first, beginning of second repeat. Final cadence with *finalis* b58 lowest pitch of the melody, change to major b59, change of register and texture b61
	170-171**	Boundary	2nd minor part, A: end of 12-bar antecedent on HC, beginning of consequent
		Phrase repetition	(repeat of antecedent), change of register to upper octave
	218-219	Boundary	2nd major part, coda: end of 2nd minor part, beginning of coda, change of key
4th	75-76	Transition	Exposition: within coda, change of texture & timbre, decrease of dynamics, modal cadence with 5-6 progression
	248-250	Boundary	Coda: beginning of *stretta* (120 bpm to 140 bpm), homophonic, clear rhythm

**Note*: no repeat. ***Note*: Bars in relation to repeats
